# Helium Enhances *Brassica napus* Cadmium Tolerance by Reducing Cadmium Uptake/Translocation and Modulating Redox Balance

**DOI:** 10.3390/antiox15101221

**Published:** 2026-09-22

**Authors:** Pengfei Cheng, Peng Xue, Weiyi Li, Jun Wang, Chenxu Cai, Rongzhan Guan, Wenbiao Shen

**Affiliations:** 1 Laboratory Center of Life Sciences, College of Life Sciences, Nanjing Agricultural University, Nanjing 210095, China; t2024051@njau.edu.cn (P.C.); 2024116075@stu.njau.edu.cn (P.X.); 2025116078@stu.njau.edu.cn (W.L.); 2023216041@stu.njau.edu.cn (J.W.); 2023216040@stu.njau.edu.cn (C.C.); 2 National Key Laboratory of Crop Genetics & Germplasm Enhancement and Utilization, Jiangsu Collaborative Innovation Center for Modern Crop Production, Nanjing Agricultural University, Nanjing 210095, China; guanrzh@njau.edu.cn

**Keywords:** *Brassica napus* L, helium-enriched solution, cadmium, uptake and translocation, ROS homeostasis

## Abstract

Helium, a traditionally inert gas, has garnered interest in medicine, yet its role in plant heavy metal responses is still elusive. Here, we investigated whether or how helium orchestrates rapeseed cadmium (Cd) tolerance. Hydroponic experimental results demonstrated that helium-enriched solution markedly alleviated Cd-induced inhibition of root growth. Importantly, helium-inhibited Cd uptake was evidenced by non-invasive micro-test technology (NMT). By using grafting experiments, we clearly observed differential responses in Cd accumulation. Since shoot tissues had relatively lower Cd contents, the inhibited root-to-shoot Cd translocation might be used to explain the low Cd accumulation achieved by helium. Further analysis indicated that helium enhanced Cd sequestration in root cell walls. Remarkably, Cd-triggered oxidative damage was effectively eliminated via stimulating the antioxidant system. Collectively, these findings suggest that restricted Cd uptake and inhibition in translocation, as well as antioxidant capacity stimulation, might be important mechanisms for helium-orchestrated Cd tolerance, thus providing a potential approach for sustainable agriculture and food security.

## 1. Introduction

Heavy metal pollution is a severe global environmental issue, posing a significant threat to agricultural systems and food safety. Normally, the exposure of natural minerals and human activities leads to the accumulation of toxic heavy metals in farmland soil [1]. Among these, cadmium (Cd) is of particular concern due to its high solubility and mobility in soil, as well as its widespread release from industrial and agricultural activities [2,3,4,5]. For crops, Cd accumulation not only severely inhibits growth and development, including damaging root architecture, disrupting photosynthesis, and causing nutrient and redox imbalance, but also enters the food chain, thus posing serious risks to human health [1]. Consequently, developing effective strategies to mitigate Cd toxicity in crops is a crucial objective for agricultural and environmental sciences.

The phytotoxicity of Cd is largely dependent on its uptake and translocation within crops [6,7]. Cd enters root cells mainly through transporters for essential elements such as silicon, zinc, and calcium [8,9,10]. Its distribution from roots to shoots is a critical process that determines the extent of aerial tissue damage. Understanding and interfering with these processes, including reducing root uptake and inhibiting long-distance translocation, especially from roots to shoots, represents a key approach for enhancing plant Cd stress tolerance [6,11,12]. Although several agronomic and genetic strategies have been explored, the discovery of novel, safe, and effective methods to block Cd entry into crops remains an ongoing pursuit.

Since the past thirty years, hydrogen (H_2_), argon (Ar), and xenon (Xe), previously regarded as the biological inertness gases, have attracted increasing interest in medical and subsequent plant sciences [13,14,15,16]. Among them, helium (He) has been demonstrated to have remarkable pharmacological properties, such as providing cardio- and neuro-protection in mammalian models [17,18,19]. Related mechanisms involve influencing reactive oxygen species (ROS) homeostasis and signaling, thus attenuating oxidative stress [17].

From a geological perspective, helium is often found as an associated gas with hydrocarbons of natural gases. In geological strata, helium concentrations could reach as high as 350–1243 ppm [20]. These data indicate that the potential biological functions of geological helium in crop physiology might not be entirely ruled out. Consistently, some recent investigations did find helium control of salinity tolerance, where both ion and redox homeostasis were re-established by helium-driven nitric oxide (NO) [21], another famous signal molecule in both animals and plants [22]. By contrast, less attention has been placed on the possible functional link between helium and heavy metal responses in crops.

Rapeseed (*Brassica napus* L.) is an economically important oil crop widely cultivated. It serves as a primary source of vegetable oil for human consumption and protein for animal feed, playing vital roles in global food security and the agricultural economy [23]. However, its production is frequently challenged by various abiotic stresses, including soil salinity and heavy metal contamination, which significantly limit its yield and quality [24,25]. In this study, we sought to understand whether or how helium positively influences crop responses upon Cd exposure, and rapeseed, an oil crop that is cultivated worldwide and ubiquitously facing great challenges from environmental cadmium stress [24,25], was used as experimental material. We comprehensively investigated the mitigating effects of helium-enriched solution on cadmium toxicity and the underlying mechanism, evaluated by changes in growth responses, Cd uptake/distribution/translocation from root to shoot, cell wall composition, and the antioxidant system. Combined with the recent findings in salinity tolerance achieved by helium [21], our results further support the proposition that chemically inert gases may not always be biologically inert, and also highlight their potential roles in stress resilience of plants and bioremediation.

## 2. Materials and Methods

### 2.1. Preparation of Helium-Enriched Solution

Helium-enriched solution was produced by bubbling purified helium gas (99.99%, *v*/*v*; Nanjing Special Gases Factory Co., Ltd., Nanjing, China) into 1 L quarter-strength Hoagland solution at a speed of 200 mL min^−1^ for 30 min, which was regarded as the saturated solution. Then, it was immediately diluted with the nutrient solution for the indicated saturation (1%, 10%, 50%, and 100%) [21]. Also, quarter-strength Hoagland solution alone (0%) was used for the control (Con) treatment.

### 2.2. Plant Materials and Growth Conditions

Uniform seeds of rapeseed (*Brassica napus* L. ‘Zhongshuang 11’) were surface-sterilized (5% NaClO), rinsed with distilled water, and soaked for 24 h in darkness. Afterwards, uniform seedlings were transferred to plastic containers (dimensions: 20 cm × 10 cm × 5 cm) containing 1 L quarter-strength Hoagland nutrient solution, and cultivated in an illuminating incubator (16 h light, 25 ± 1 °C/8 h dark, 20 ± 1 °C).

Three-day-old rapeseed seedlings were pretreated (lasting for 1 day before stress), co-treated (lasting for 3 days with stress), or post-treated (lasting for 1 d, after stress-removed) with the indicated helium-enriched solutions (detailed illustrated in Figure 1). The final concentration of 10 μM cadmium chloride (CdCl_2_) was used for the stress condition [26]. The nutrient solution was renewed every 24 h to maintain nutrient stability and helium content. All experiments were performed using three independent biological replicates. Three containers per biological replicate were assigned for each treatment. For each container, 120 seedlings were applied. After treatments, unless otherwise stated, seedlings in one container were mixed and randomly sampled for analysis immediately, or samples were stored at −80 °C for further analysis.

### 2.3. Grafting Experiment

Grafted seedlings were obtained from a previous method [27]. After being pretreated with helium for 1 day, four grafted combinations were generated immediately, including control shoot/control root (CSCR), helium-pretreated shoot/control root (HeSCR), control shoot/helium-pretreated root (CSHeR), and helium-pretreated shoot/helium-pretreated root (HeSHeR). Then, the four grafted combinations were used for the stress experiment for 3 days (detailed illustrated in Figure 1).

### 2.4. Analyses of Oxidative Damage Assay

Thiobarbituric acid reactive substances (TBARS) were analyzed by spectrophotometry [28]. The relative electrical conductivity (REC) was analyzed by an electronic conductivity meter (DDS-12A; Kangyi Instrument, Shanghai, China) [29].

Hydrogen peroxide (H_2_O_2_) and superoxide anion (O_2_^.−^) contents were measured by both spectrophotometry and histochemical methods.

In brief, H_2_O_2_ content was measured by the spectrophotometry with a minor modification. After the assay reagent was incubated for 45 min, the absorbance of the Fe3^+^-xylenol-orange complex was determined at 560 nm. A standard curve was obtained by adding variable amounts of H_2_O_2_ [29]. The roots were also stained with 3,3′-diaminobenzidine (DAB) for histochemical H_2_O_2_ content analysis [30].

For O_2_^.−^ content, 0.2 g tissue was ground within 2 mL of sodium phosphate buffer (50 mM, pH 7.8) for homogenization in an ice bath. After centrifugation at 12,000× *g* and 4 °C for 15 min, the mixture containing the supernatant was then combined with 1 mL of sodium phosphate buffer (50 mM, pH 7.8) and hydroxylammonium chloride (10 mM) before a 20 min reaction at 25 °C. The obtained solution was then combined with 1 mL of 4-aminobenzene sulfonic acid (17 mM) and α-naphthylamine (7 mM) for another 20 min reaction at 25 °C. Its optical density at 530 nm was then recorded [29]. The roots were also stained with nitroblue tetrazolium (NBT) for histochemical O_2_^.−^ analysis [30].

### 2.5. Determination of Cd Content

Besides whole plants, xylem saps and subcellular components, including cell wall fractions, cell soluble fractions, and organelle fractions, were separated and then used for the following Cd content determination [26,31].

Samples were oven-dried at 65 °C until a constant dry weight and then digested by the microwave digital digestion system (Milestone Ethos T, Sorisole, Italy). The concentrations of Cd were determined using Inductively Coupled Plasma Mass Spectrometry (ICP-MS; NexION 8000, Perkin Elmer, Waltham, MA, USA) [31].

### 2.6. Net Influx of Cd

Cd fluxes in the root were measured using non-invasive micro-test technology (NMT; NMT-YG-100, Younger USA LLC, Amherst, MA, USA) following established protocols. The Cd influx of instantaneous and steady states, which was distinguished according to the variation tendency [26], was analyzed in the elongation zone of root tips.

### 2.7. Determination of Antioxidant Enzyme Activities

For the assay of antioxidant enzymes, the activities of ascorbate peroxidase (APX), superoxide dismutase (SOD), catalase (CAT), and peroxidase (POD), were estimated according to the previous methods [29,30,32]. For SOD activity, one unit (U) was defined as the amount of inhibition of NBT photo-reduction. The above enzyme activities were normalized to the protein content determined using the BCA Protein Quantification Kit (Vazyme, Nanjing, China).

### 2.8. RT-qPCR Analysis

Relative expression levels of corresponding genes were presented as values relative to those of corresponding control samples, after normalization with two reference genes, *BnActin* and *BnGAPDH*. Detailed information about gene-specific primers can be found in Supplemental Table S1. The results were analyzed relative to gene expression levels using the 2^−ΔΔCT^ method [33].

### 2.9. Statistical Analysis

Statistical analysis was conducted using Origin 2025. Data were presented as mean ± SD. The significance of differences between data sets was determined using one-way ANOVA followed by Duncan’s multiple comparison (*p* < 0.05) or Student’s *t*-test. For comparisons between two groups, Student’s *t*-test was applied. When experiments involved three or more treatment groups, one-way ANOVA followed by Duncan’s multiple comparison test was used to determine significant differences among means. The specific statistical test used for each experiment is also indicated in the corresponding figure legends.

## 3. Results

### 3.1. Cd-Induced Growth Inhibition in Response to Helium

Since helium has slightly diffusivity and a high escaping tendency [34,35], helium-enriched nutrient solution was herein utilized [21]. To best identify the optimal and effective treatments, cadmium-stressed rapeseed seedlings were subjected to pretreatment, cotreatment, or post-treatment with nutrient solutions enriched with helium at 0% (Con), 1%, 10%, 50%, and 100% saturation levels (Figure 1).

After 3 days of Cd exposure and 1 day recovery, a pronounced inhibition of rapeseed seedling growth was observed in the stressed alone group (*p* < 0.05; Figure 2). For pretreatment and cotreatment, unlike the weak and no significant responses achieved by 1%, 10%, and 100% helium-enriched solutions, only the 50% helium-enriched solution significantly alleviated the reduction in fresh weight caused by Cd. For post-treatment groups, however, all helium addition groups failed to influence the growth stunt. It was noteworthy that in the absence of Cd stress, none of the above helium treatments had a significant impact on seedling growth performance, regardless of their concentrations or modes of application. The above results clearly reflected the possibility that helium acts specifically under the stress conditions, and pretreatment with 50% helium-enriched solution was adopted for the subsequent investigation.

### 3.2. Cd-Stressed Performances Were Rescued by Helium

As expected, Cd stress not only significantly inhibited seedling growth (Figure 3A), but also negatively influenced membrane damage in root tissues, evidenced by the increase in relative electrolyte leakage (REC; Figure 3B) and thiobarbituric acid-reactive substance (TBARS; Figure 3C) accumulation. Helium obviously reduced membrane damage, showing obvious decreases in REC (28.4 ± 3.6% and 30.4 ± 4.6%) compared to those in Cd-stressed alone roots and shoots (36.3 ± 3.1% and 36.8 ± 6.1%). These results clearly demonstrated that the alleviated membrane damage might be used to explain Cd stress resilience of plants achieved by helium.

### 3.3. Inhibited Root-to-Shoot Cd Translocation by Helium

We clearly observed that Cd accumulation in shoots, roots, and xylem sap was remarkably decreased by ~47.3%, 11.7%, and 20.5% in the presence of helium pretreatment (Figure 4A–C).

For addressing related mechanisms, grafting experiments were adopted. After helium pretreatment, four graft combinations were immediately adopted (Figure 4D), including control shoot/control root (CSCR), helium-pretreated shoot/control root (HeSCR), control shoot/helium-pretreated root (CSHeR), and helium-pretreated shoot/helium-pretreated root (HeSHeR). The above combinations were used to elucidate whether helium-reduced Cd accumulation was functionally linked to coordinated uptake and/or root-to-shoot translocation mechanisms.

After grafting, Cd accumulation analysis revealed that pretreatment with helium, regardless of whether applied to roots (CSHeR; especially) or shoots (HeSCR), remarkably reduced Cd accumulation in the shoot parts (Figure 4E). Unlike the contrasting response achieved by root pretreatment (CSHeR), we clearly observed that when only the shoot was pretreated with helium (HeSCR), root Cd accumulation still appeared (Figure 4F). It might be caused by the fact that Cd translocation rather than uptake/accumulation in roots was inhibited by shoot pretreatment, reflecting the importance of helium-treated locations. Consistently, contrasting the responses of the CSHeR group, a slight but no significant reduction of Cd accumulation in whole plants was observed after pretreatment with helium (HeSCR), compared to Cd stress alone (CSCR; Figure 4G). When whole plants were pretreated with helium (HeSHeR), a maximal inhibited shoot Cd accumulation was observed, accompanied by a relatively weaker inhibition in root parts, compared to those in the CSHeR group. Therefore, we speculated that the reduction in root Cd accumulation might not only be caused by helium-inhibited Cd uptake in roots, but also severely influenced by the reduced root-to-shoot translocation in both shoots and roots, as evidenced by Cd content in xylem sap (Figure 4C). The identical maximal Cd reduction in whole plants of CSHeR and HeSHeR groups matched the above speculation, confirming helium-inhibited Cd accumulation through coordinately influencing root uptake and/or root-to-shoot translocation mechanisms. The above results were also partially certified by the shoot and root gene expression of Cd translocation and tissue distribution, including *IRT1*, *HMA2*, *NRAMP1*, *NRAMP4*, and *NRAMP5* (Figure S1) [36,37,38].

### 3.4. Root Cd Uptake Was Reduced by Helium

To further confirm the above speculation, the non-invasive micro-test technology (NMT) was employed to monitor real-time Cd flux in roots of rapeseed seedlings. As anticipated, both the instantaneous and steady states of Cd influx were reduced after helium pretreatment, compared to those in chemical-free controls (Figure 5A,B). Subsequent results showed that helium notably altered the partitioning of Cd within root cells, evidenced by significantly decreased Cd accumulation in the soluble fraction and the organellar fraction, contrasting the increased Cd levels in the cell wall fraction (Figure 5C,D). These findings indicate that, besides influencing Cd distribution in root cells, helium-driven Cd accumulation inhibition might be mainly caused by reduced Cd influx in roots. These results reflected the complexity of the Cd tolerance mechanism driven by helium.

### 3.5. Redox Balance Was Enzymatically Re-Established by Helium

Histochemical and biochemical analyses were further conducted to assess whether and how helium influenced hydrogen peroxide (H_2_O_2_) and superoxide anion (O_2_^.−^) accumulation upon Cd stress. Under chemical-free conditions, helium alone did not significantly alter the endogenous ROS levels (Figure 6A–D and Figure 7A,B), as well as the activities of antioxidant enzymes in roots, including ascorbate peroxidase (APX), superoxide dismutase (SOD), catalase (CAT), and peroxidase (POD) (Figure 6E–H and Figure 7C–F). We also observed that corresponding transcripts (POD, CAT, Cu/Zn-SOD, Mn-SOD, and APX) displayed similar tendencies (Figure 6I).

Under Cd stress conditions, remarkable ROS accumulation was observed in both roots (Figure 6A,B) and shoots (Figure 7A,B), as evidenced by spectrophotometry methods and histochemical analyses, showing intense staining with DAB and NBT for H_2_O_2_ and O_2_^.−^ accumulation in roots (Figure 6C,D), reflecting redox imbalance in Cd-stressed conditions. The above results could be further supported by the inhibited activities of antioxidant enzymes, as well as their down-regulated transcripts (Figure 6E–I and Figure 7C–G). However, the above changes were obviously abolished after helium pretreatment, suggesting that re-establishing redox balance achieved by the stimulation of the enzymatic antioxidant system might be an important mechanism underlying stress resilience of plants driven by helium.

## 4. Discussion

In the last fifteen years, helium has emerged as a potentially therapeutic gas in animals [17,19]. By contrast, whether this inert gas acts as a regulator of plant responses to environmental stress has been less clear. In this study, using a combination of physiological, molecular, pharmacological, and cell biology approaches, we have unraveled helium control of Cd resilience in plants (Figure 2 and Figure 3). Although the physical and chemical alterations in helium-enriched solution (particularly dissolved O_2_ and N_2_ concentrations were influenced) could not be easily ruled out, multiple mechanisms, including influencing root uptake and/or root-to-shoot translocation, as well as reinforcing the enzymatic antioxidant system to re-establish ROS homeostasis, were mainly involved in the helium-driven response against cadmium toxicity. More importantly, it should be noticed that the effective concentration of helium pretreatment might be limited to a relatively narrow range (50% was most effective, approximately 4.3 mL/L, theoretically [39]). For this non-linear response, similar observations and complex mechanisms were previously elucidated in H_2_ and CH_4_ responses in plants [40,41], possibly reflecting complicated diffusion patterns achieved by gaseous molecules in various plant tissues [42]. Collectively, combined with our recent findings that helium orchestrated salinity stress in alfalfa plants via nitric oxide (NO) signaling [21], we speculated that helium might have myriad roles in plant responses against multiple abiotic stresses, and intensive cellular metabolic reprogramming could be influenced to fulfill the functions of helium.

The primary constraint imposed by Cd toxicity is excessive uptake and following Cd accumulation [6,9,38]. Plants adopt various cellular and molecular mechanisms to minimize Cd toxicity. Upon exposure to Cd, they initially implement avoidance strategies, such as redistributing and/or immobilizing Cd to prevent metal entry into root cells [43]. Herein, we observed that helium pretreatment effectively reduces the net influx of Cd into roots (Figure 5A,B). This immediate effect on Cd uptake represents a first and crucial barrier against toxicity [11,12,38]. More importantly, subsequent subcellular fractionation analysis revealed a strategic redistribution of Cd within root cells, especially observed binding Cd to cell walls (Figure 5C,D). This shift clearly indicates that Cd uptake was inhibited by helium to alleviate Cd toxicity in roots and shoots, which was achieved by effectively immobilizing Cd in cell wall fractions and preventing its interaction with vital metabolic sites in the soluble and organelle fractions, a well-known mechanism underlying Cd toxicity alleviation [31,44].

Besides reducing Cd accumulation in roots and shoots (Figure 4A,B), grafting experiments, an important approach for analyzing mechanisms [45], further demonstrated that helium responses are functionally linked to coordinated mechanisms involving both root and shoot tissues. Pretreating roots alone (CSHeR) significantly inhibited Cd uptake into the roots, thereby decreasing shoot and whole plant Cd contents (Figure 4D–G). Considering the decreased Cd contents in xylem saps (Figure 4C), the inhibited root-to-shoot Cd translocation in the CSHeR group was reasonable. In contrast, pretreating shoots alone (HeSCR) had a different influence on root Cd uptake, evidenced by unchanged Cd accumulation in whole plants, but effectively restricted root-to-shoot translocation, potentially involving altered distribution, vacuolar sequestration, or reduced translocation within the shoot [45,46,47]. When both tissues were pretreated (HeSHeR), the highest inhibition of shoot Cd accumulation was clearly observed, indicating that helium inhibits Cd accumulation through coordinately influencing root uptake and root-to-shoot translocation (especially). Molecular evidence also confirmed that the gene expressions of Cd transport were decreased by helium pretreatment (Figure S1). This complex interplay suggests that helium triggers coordinated responses in root and shoot tissues, thus effectively minimizing Cd toxicity in shoots, the sensitive tissue upon Cd stress, compared to roots [47].

Ample evidence confirms that Cd exposure increases the overproduction of ROS, thus resulting in redox imbalance in plant cells [17,19]. The overaccumulation of ROS could damage the plasma membrane, and upon Cd stress, plants have developed an efficient antioxidant system to eliminate ROS and protect their tissues and cells [48,49,50]. Beyond impacting Cd distribution and accumulation, our further results revealed that helium plays a vital role in counteracting the oxidative stress in rapeseed triggered by Cd, and a similar mechanism was elucidated in enhanced alfalfa salinity tolerance achieved by helium [21]. For example, under Cd-free conditions, there was no discernible effect of helium on ROS content and the antioxidant system (Figure 6 and Figure 7), indicating that helium did not obviously perturb metabolism. Upon Cd exposure, helium was found to be highly effective in alleviating oxidative damage (Figure 6A–D and Figure 7A,B), assessed by significant decreased REC (Figure 3B) and TBARS content (Figure 3C). This protective effect is functionally linked to alleviating oxidative damage [51], including elevating the enzymatic antioxidant system (Figure 6E–H and Figure 7C–G) or reducing Cd accumulation (Figure 4 and Figure 5), either directly or indirectly. These responses were further confirmed by corresponding transcripts of antioxidant genes (Figure 6I and Figure 7G) and Cd transport genes (Figure S1).

In this study, treatments were applied at the seedling stage with a 3-day exposure, representing a critical developmental period highly susceptible to Cd stress [52,53]. Different growth stage exposure may yield different outcomes, and this possibility warrants further investigation. Notably, the protective mechanisms illustrated herein are not limited to Cd toxicity, but might be applied to other heavy metal exposures. Certainly, the individual and synergistic contribution caused by helium-influenced NO signaling, ROS production, sensing, and signaling should be validated [21,54].

## 5. Conclusions

In conclusion, this study provides a foundational understanding of helium as a regulator for enhancing stress resilience of plants, especially Cd toxicity. A mechanistic model whereby helium orchestrates multi-faceted defense strategies was proposed. First, Cd accumulation was inhibited by helium through coordinately influencing root uptake and root-to-shoot translocation mechanisms. Second, helium helps to fortify the root physiological barriers against Cd uptake, thus promoting its immobilization in cell walls. Third, the enzymatic antioxidant system was stimulated, thus alleviating Cd-induced oxidative damage. These findings open a new window for helium-based biology in plants, positioning it as a promising and potentially sustainable tool for phytoremediation and agricultural management in Cd-polluted soils.

## Figures and Tables

**Figure 1:**
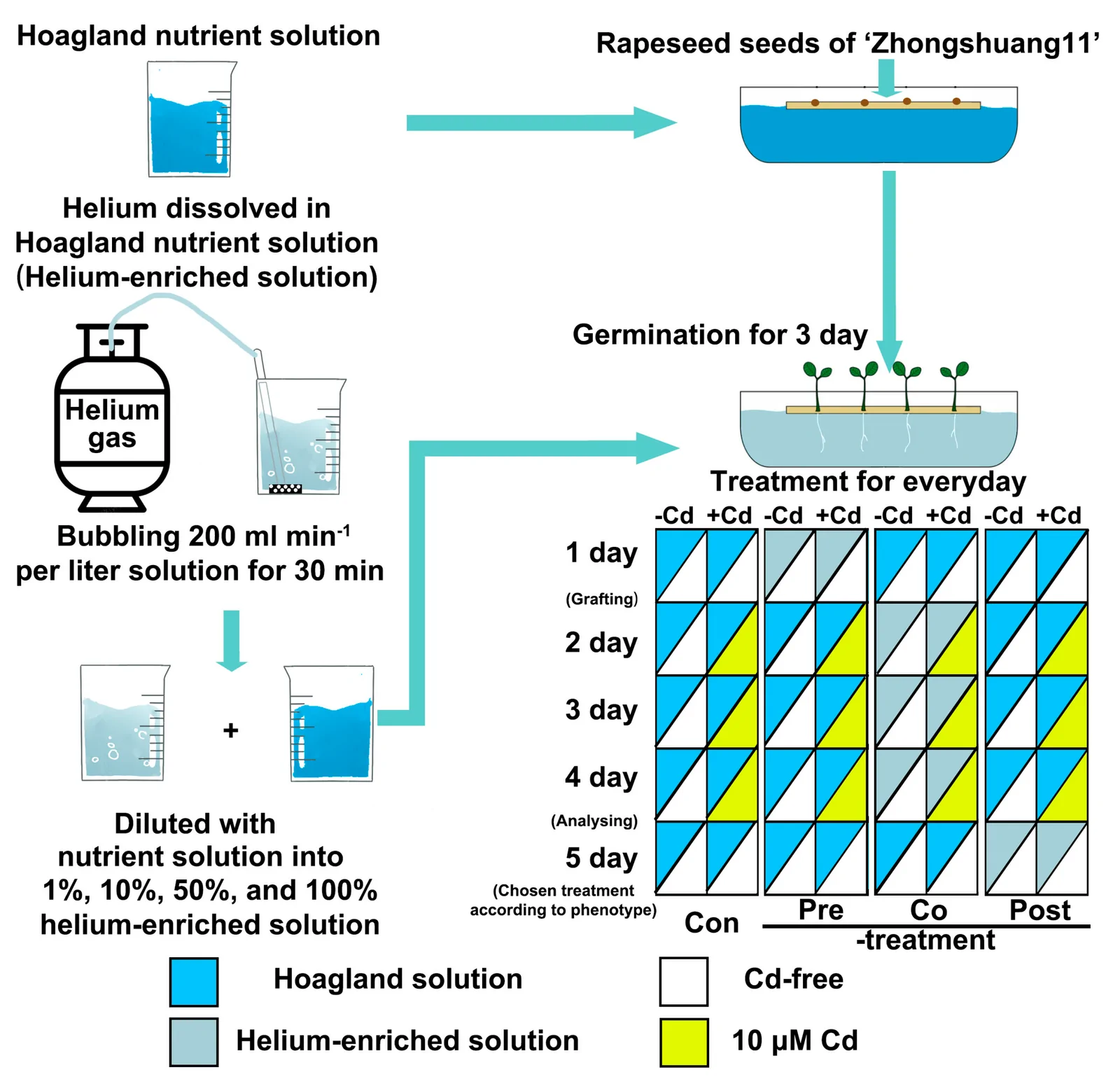
A framework of experimental design used in the study. High-purity (99.99%) helium gas was dissolved in the 1/4 Hoagland nutrient solution through the bubbling approach (200 mL min^−1^ gas bubbling into 1/4 Hoagland nutrient solution per liter for 30 min). Then, the stock solution was immediately diluted with the nutrient solution for the indicated saturation. The final concentration of 10 μM cadmium chloride (CdCl_2_) was used for the stress experiment for 3 d. Three-day-old rapeseed seedlings were pretreated (lasting for 1 day before stress), co-treated (lasting for 3 days with stress), or post-treated (lasting for 1 d after stress removed) with the indicated helium-enriched solutions. The seedlings were grafted after pretreatment (4 days after germination), and used for analysis after Cd stress for 3 d.

**Figure 2:**
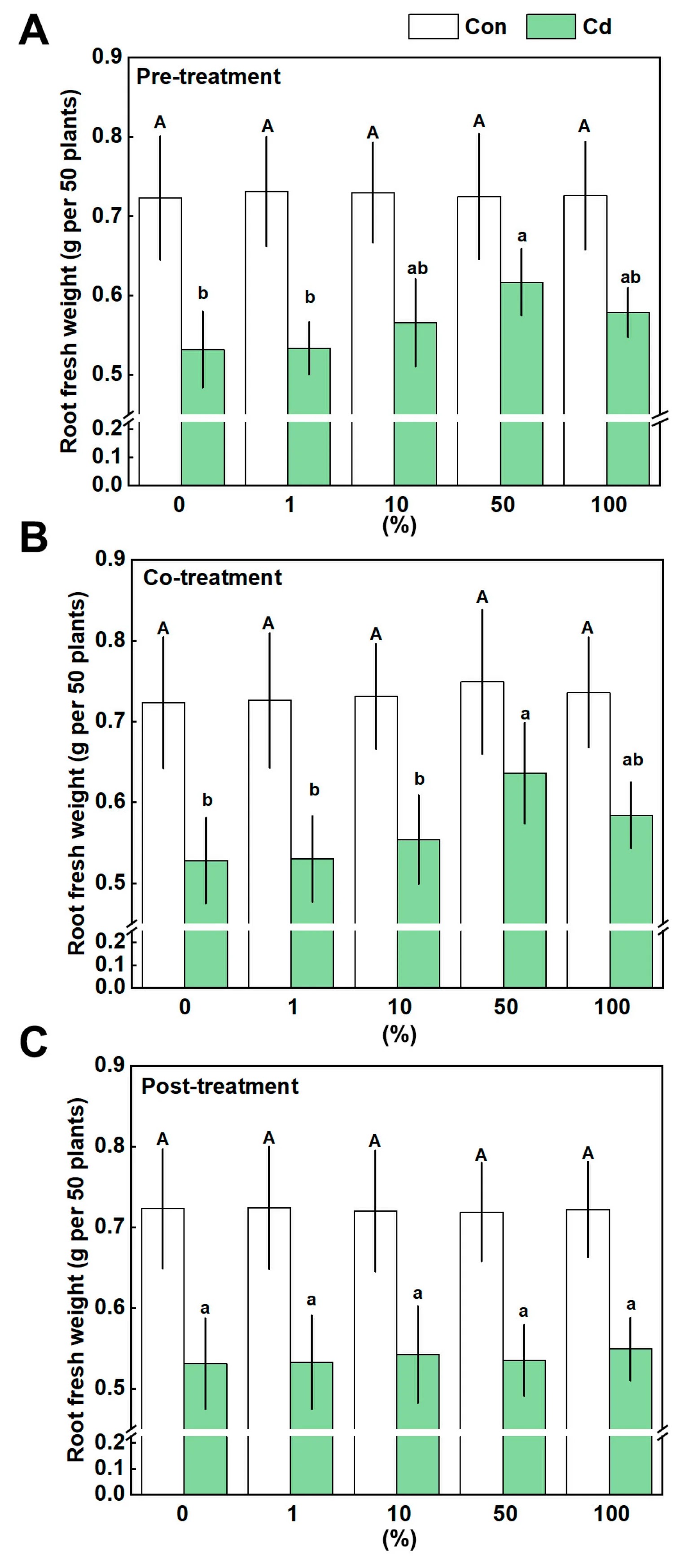
Pretreatment with helium was chosen to confirm helium functioning in stress resilience against Cd exposure. Three-day-old rapeseed roots were hydroponically pretreated (**A**), co-treated (**B**), and post-treated (**C**) experiments with the indicated helium-enriched solutions (%), as illustrated in Figure 1. After 5 days, fresh weight was determined. Bars with different letters are significantly different at *p* < 0.05 (replication = 3, 50 plants/sample/treatment/replication) in accordance with one-way ANOVA followed by Duncan’s multiple comparison test.

**Figure 3:**
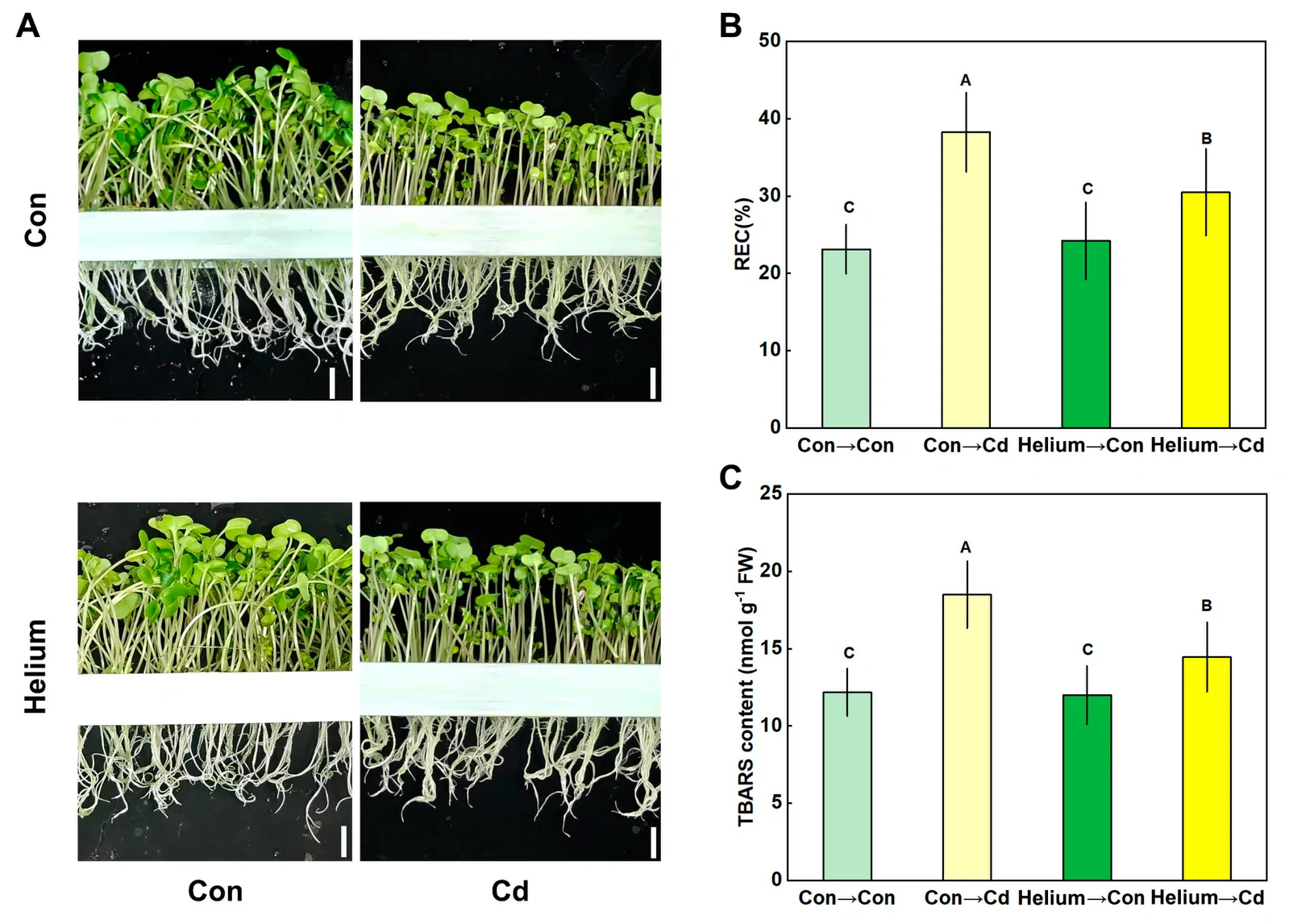
Altered Cd-stressed performances were rescued by helium. After 1 day of pretreatment with 0 (Con) or 50% helium-enriched solution (Helium), followed by 0 (Con) or 10 μM CdCl_2_ exposure (Cd) for 3 days, phenotypic photos ((**A**), bars = 1 cm), relative electrical conductivity (REC; (**B**)), and TBARS content (**C**) in roots were recorded. Bars with different letters were significantly different at *p* < 0.05 (n = 3 replication, 0.2 g FW/sample/treatment/replication) in accordance with one-way ANOVA followed by Duncan’s multiple comparison test.

**Figure 4:**
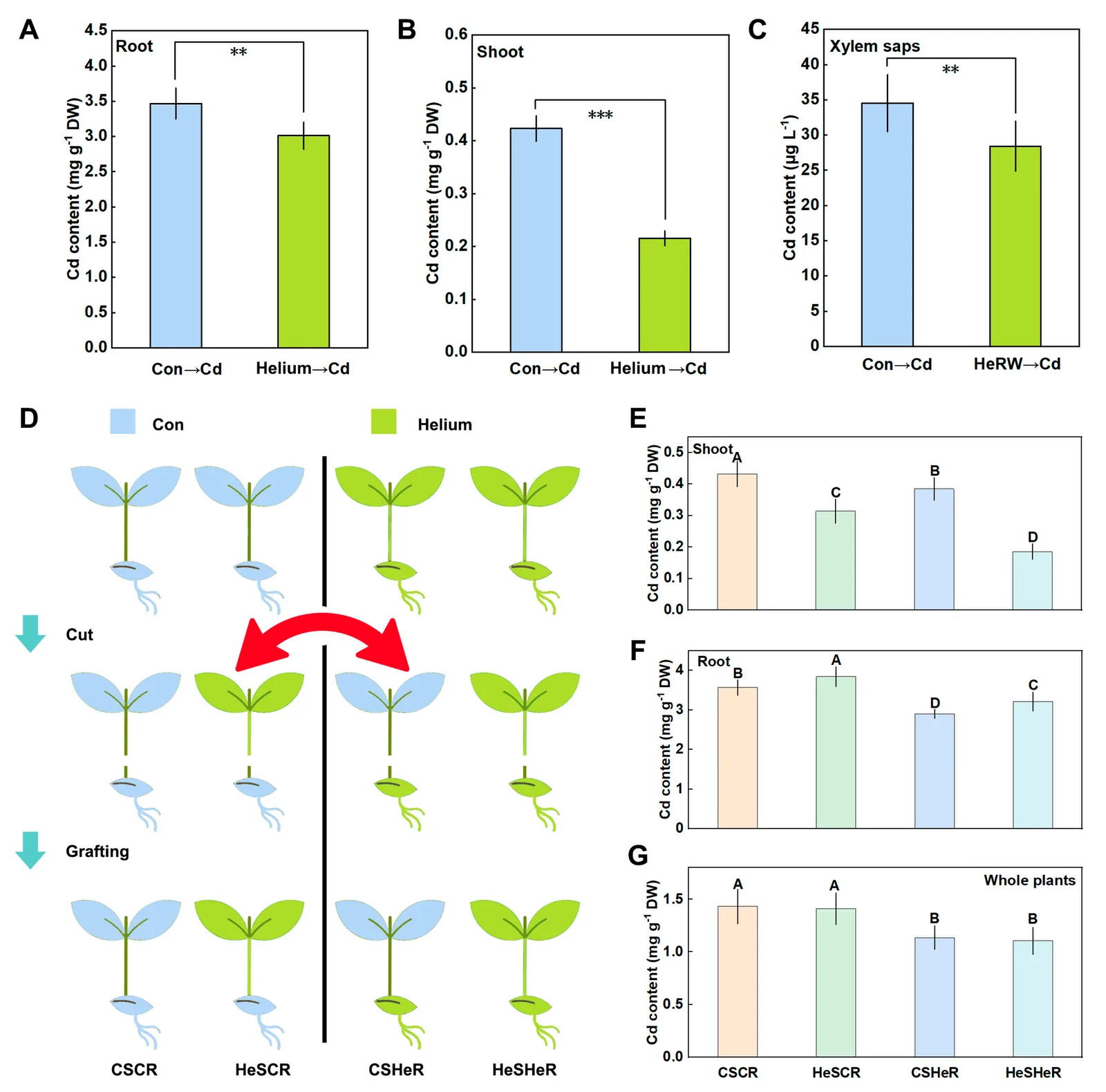
Grafting experiments confirm that helium-decreased Cd accumulation was achieved by impairing root-to-shoot translocation. After helium pretreatment followed by Cd stress for 3 days, Cd contents in roots (**A**), shoots (**B**), and xylem saps (**C**) were determined. Asterisks represent significant differences by Student’s *t*-test (** *p* < 0.01 and *** *p* < 0.001). For addressing its mechanism, graft experiments were adopted (**D**), as illustrated in Figure 1. After being pretreated with 0% (Con, blue color) or 50% helium-enriched solution (Helium, green color), four grafted combinations were generated immediately, including control shoot/control root (CSCR), helium-pretreated shoot/control root (HeSCR), control shoot/helium-pretreated root (CSHeR), and helium-pretreated shoot/helium-pretreated root (HeSHeR). The red arrow indicated that the shoots were exchanged and then grafted. After grafting for 3 days, shoot (**E**), root (**F**), and whole plant (**G**) Cd contents were analyzed. Bars with different letters are significantly different at *p* < 0.05 in accordance with one-way ANOVA followed by Duncan’s multiple comparison test. n = 5 replication, and 0.2 g DW/sample/treatment/replication.

**Figure 5:**
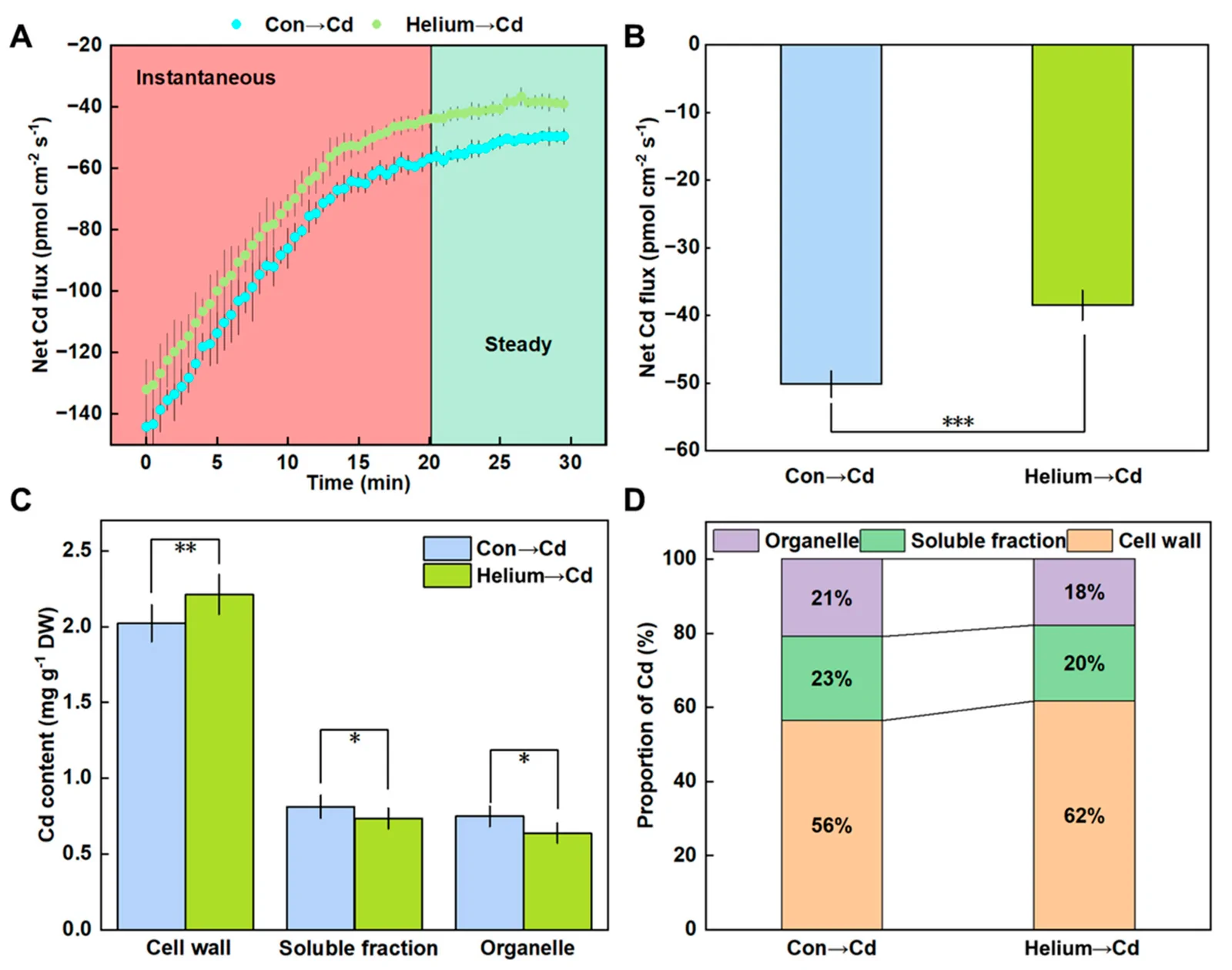
Root Cd uptake was inhibited by helium. After 1 day of pretreatment with 0 (Con) or 50% helium-enriched solution (Helium), the instantaneous and steady states of Cd influx in root tips ((**A**,**B**); at the beginning of Cd exposure; *n* = 3 technical repetitions) were analyzed by non-invasive micro-test technology (NMT) system. After 0 (Con) or 10 μM CdCl_2_ exposure (Cd) for 3 days, Cd contents (**C**) and proportion (**D**) of different root cell fractions (*n* = 3 replication, 0.05 g/sample/treatment/replication) were analyzed. Asterisks represent significant differences by Student’s *t*-test (* *p* < 0.05, ** *p* < 0.01, and *** *p* < 0.001).

**Figure 6:**
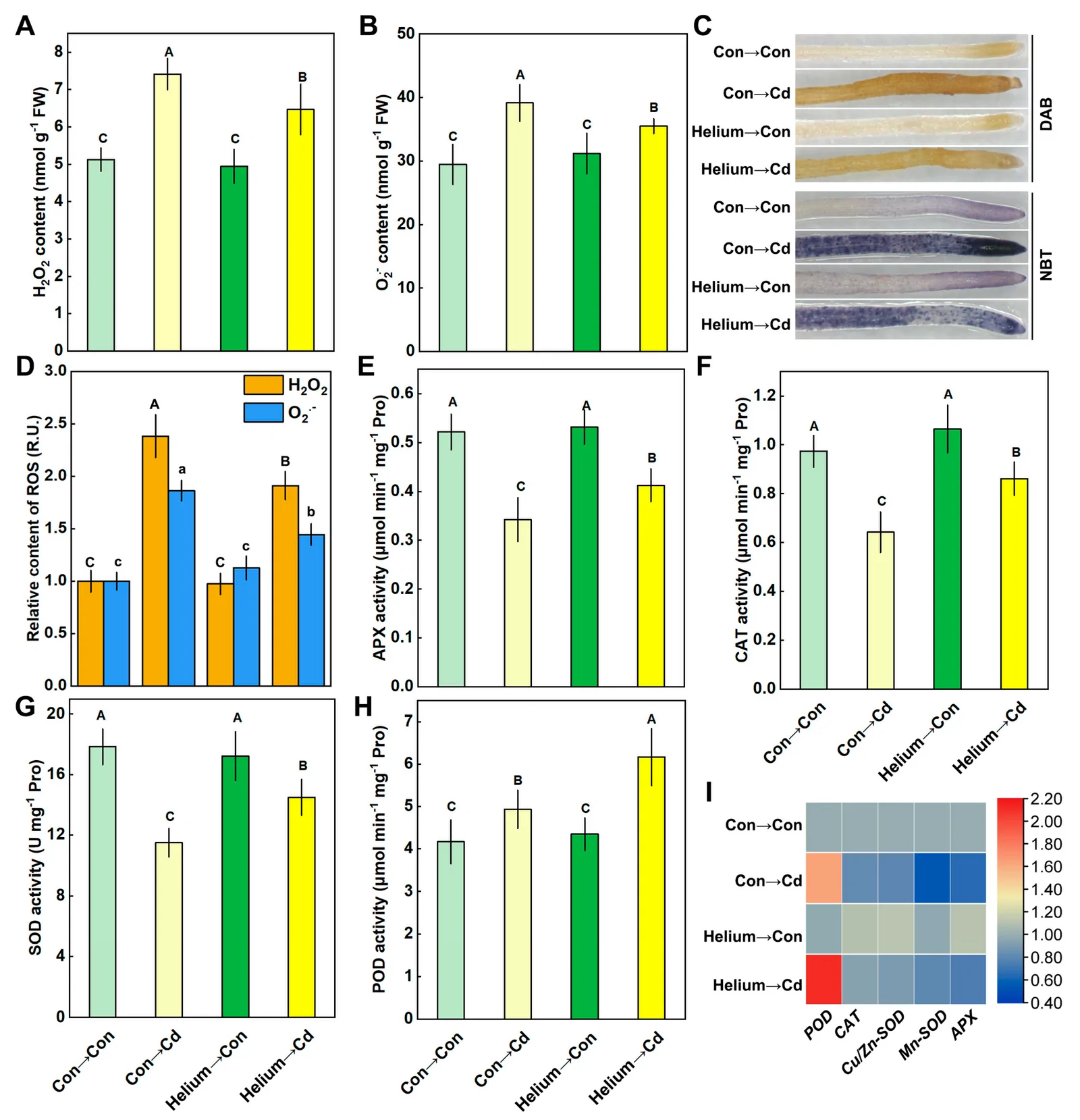
Redox homeostasis was enzymatically re-established by helium. After helium pretreatment followed by Cd stress for 3 days, the contents of H_2_O_2_ (**A**) and O_2_^.−^ (**B**) in roots were analyzed by spectrophotometry and staining with 3,3′-diaminobenzidine (DAB; (**C**), upper) and nitroblue tetrazolium (NBT; (**C**), bottom), respectively. The intensity of DAB and NBT staining was quantified by gray-scale analysis using ImageJ software (1.53). The relative staining intensity was calculated as the integrated density value normalized to the root area, and data are presented as relative H_2_O_2_ and O_2_^.−^ content (**D**). The activities of ascorbate peroxidase (APX; (**E**)), catalase (CAT; (**F**)), superoxide dismutase (SOD; (**G**)), and peroxidase (POD; (**H**)), as well as corresponding transcripts (*POD*, *CAT*, *Cu/Zn-SOD*, *Mn-SOD*, and *APX*) in roots were analyzed (**I**). Bars with different letters are significantly different at *p* < 0.05 (n = 3 replications, 3 roots or 0.2 g FW/sample/treatment/replication; 3 roots/treatment/replication for intensity analysis) in accordance with one-way ANOVA followed by Duncan’s multiple comparison test.

**Figure 7:**
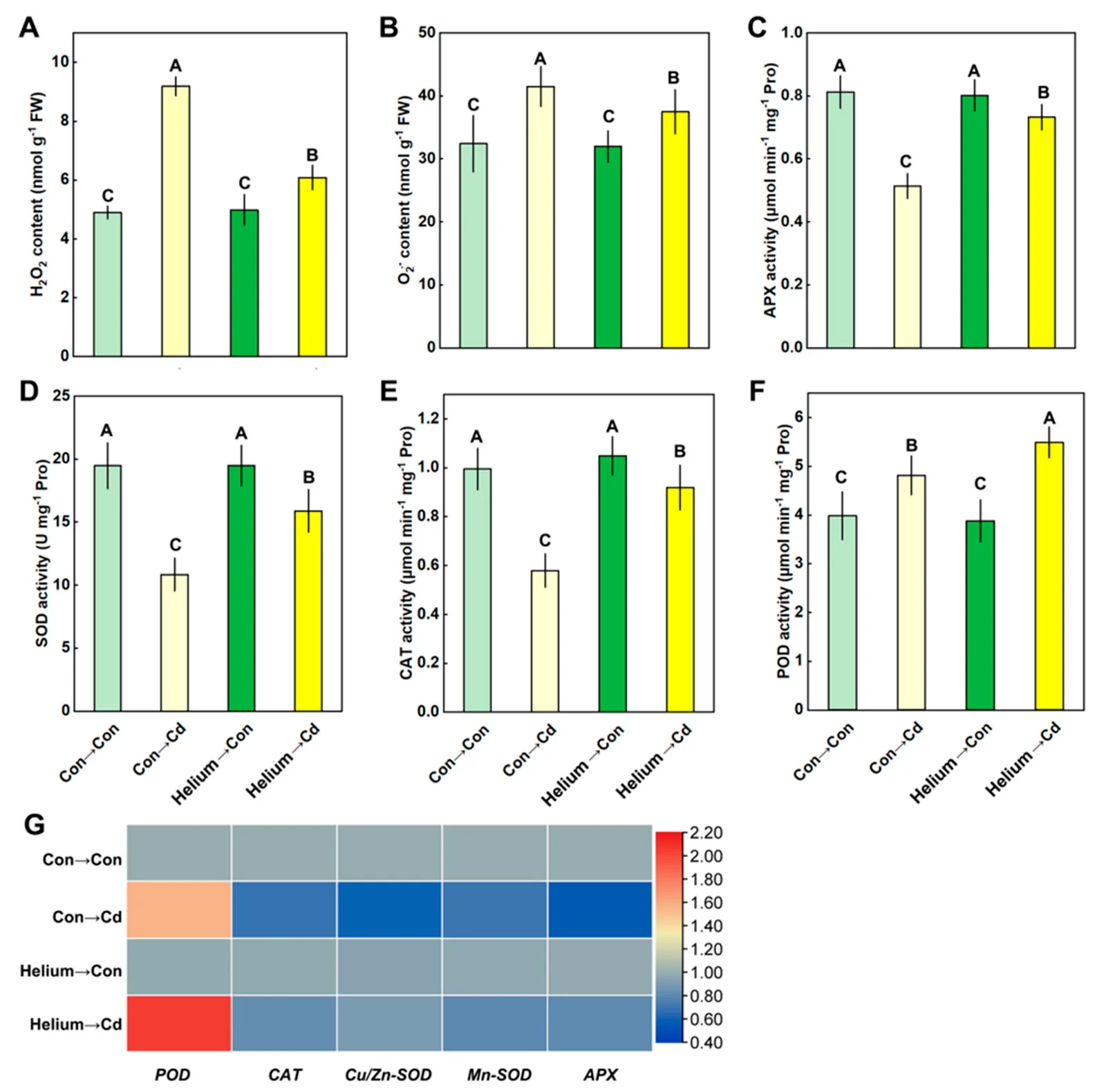
Redox homeostasis in shoots was regained following helium treatment. After helium pretreatment followed by Cd stress for 3 days, the contents of H_2_O_2_ (**A**) and O_2_^.−^ (**B**) in shoots were analyzed by spectrophotometry. The activities of ascorbate peroxidase (APX; (**C**)), superoxide dismutase (SOD; (**D**)), catalase (CAT; (**E**)), and peroxidase (POD; (**F**)), as well as corresponding transcripts (*POD*, *CAT*, *Cu/Zn-SOD*, *Mn-SOD*, and *APX*) in shoots were analyzed (**G**). Bars with different letters are significantly different at *p* < 0.05 (n = 3 replications, 0.2 g FW/sample/treatment/replication) in accordance with one-way ANOVA followed by Duncan’s multiple comparison test.

## Data Availability

No new data were created or analyzed in this study. Data sharing is not applicable to this article.

## Supplementary Materials

The following supporting information can be downloaded at https://www.mdpi.com/article/10.3390/antiox15101221/s1: Table S1. The sequences of primers for qPCR; Figure S1. Cd accumulation decrease was achieved by inhibiting transcripts profile of Cd transport gene expression. After helium pretreatment followed by Cd stress for 3 days, the transcripts (*IRT1*, *HMA2*, *NRAMP1*, *NRAMP4*, and *NRAMP5*) in shoots (A) and roots (B) were analyzed by RT-qPCR.
