# Supplementary material for: Helium Enhances Brassica napus Cadmium Tolerance by Reducing Cadmium Uptake/Translocation and Modulating Redox Balance
Source: Antioxidants (Basel). 2026 Sep 22;15(10):1221. doi: 10.3390/antiox15101221 (PMC13700796; doi:10.3390/antiox15101221)
Supplement: Supplementary file 1 [file antioxidants-15-01221-s001.zip › antioxidants-4491838-supplementary.pdf]

Supplementary Table S1 The sequences of primers for qPCR

Gene name	Accession number	Forward primer	Reverse primer
GAPDH	XM_009125769.3	TTGGTGACAACAGGTCAAGCA	AAACTTGTCGCTCAATGCAATC
Actin	XM_009127096.3	TGTGCCAATCTACGAGGGTTT	TTTCCCGCTCTGCTGTTGT
CAT	XM_013797971.2	GCCGAACCCGAAAACAAA	GTCATCAAACATCCAGCACCA
Cu/Zn-SOD	XM_033281479.1	TCACTGACAGCCAGATTCCTC	CCTGCGTTTCCAGTAGACAA
Mn-SOD	XM_009148545.3	TGGAAGCGTCTCCCTTGTTC	TTTCTCGGCCCATCTACGTG
POD	XM_013859762.2	CTCCCACCGTATCCTCG	CGTGCTGCTCAAAGTCG
APX	XM_009149789.3	CCCATTCGGAACAATGAGGT	ACAGCCACAACACCAGCAAG
BnNRAMP1;1	BnaA02T0239800	AAACGTGGTGGGTAAATGGA	TCACGTATTGCCCAGCATAA
BnNRAMP4;1	BnaA07T0140800	TCGCTGTTATCCCTCTGCTT	CACCAGCAAAACCACACTTG
BnNRAMP5;2	BnaC07T0425700	TCTTCAAAATCGGACCTTCG	CTGCAGCCATGAAGAAATCA
BnHMAP2;3	BnaC01g08300D	TGCTGTGAAAGTTCAAGGAAAG	ACTGTGACAGTGACTATGACTG
BnHMAP2;6	BnaAnng30640D	GCAATGTTAACCGGAGATAACC	TTGGACCTTCTCTCTTGAACTC
BnIRT1;1	BnaA01g35000D	GATACTTGCACCATCAAAGGAC	ACTGCATTTTGATGCTACCTTG
BnIRT1;2	BnaA01g35020D	CAATCTCTCCAGCAACTTCAAC	ACCAATCATGCTTGCTACTAGT

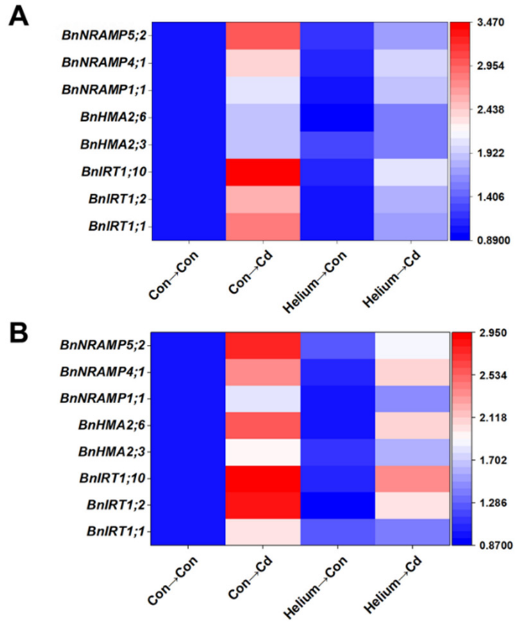

Figure S1. Cd accumulation decrease was achieved by inhibiting transcripts profile of Cd transport gene expression. After helium pretreatment followed by Cd stress for 3 days, the transcripts (*IRT1*, *HMA2*, *NRAMP1*, *NRAMP4*, and *NRAMP5*) in shoots (A) and roots (B) were analyzed by RT-qPCR.
